# Using Photovoice to stimulate critical thinking: An exploratory study with Nursing students*

**DOI:** 10.1590/1518-8345.3625.3314

**Published:** 2020-07-01

**Authors:** Elena Andina-Díaz

**Affiliations:** 1 University of León, Nursing and Physiotherapy Department, Vegazana Campus, León, Spain.; 2 University of León, Faculty of Health Sciences, SALBIS Research Group, León, Spain.; 3 University of Alicante, Faculty of Health Sciences, Nursing and Culture of Care Research Group (EYCC), San Vicente del Raspeig, Alicante, Spain.

**Keywords:** Qualitative Research, Nursing Education, Methods, Teaching Materials, Photography, Thinking, Pesquisa Qualitativa, Educação em Enfermagem, Métodos, Materiais de Ensino, Fotografia, Pensamento, Investigación Cualitativa, Educación en Enfermería, Métodos, Materiales de Enseñanza, Fotografía, Pensamiento

## Abstract

**Objective:**

to explore the potentialities of the Photovoice methodology to stimulate critical thinking on Social Determinants of Health.

**Method:**

an exploratory and descriptive study with a qualitative approach, using different steps of the Photovoice methodology. Nursing students obtained photographs in their community, showing Social Determinants of Health, analyzed and classified the photographs, and exposed the results in the Nursing school. The students answered a questionnaire writing their perceptions. The data collected from the questionnaires were qualitatively analyzed.

**Results:**

91 students participated in the study. Two main categories emerged from the data: Photovoice is a good methodology to stimulate critical thinking on Social Determinants of Health, and Photovoice is a good methodology to stimulate other skills (expressing beliefs and perceptions, stimulating creativity, developing research skills, strengthening ties with colleagues, and attracting attention).

**Conclusion:**

we explore the potentialities of the Photovoice methodology. It can be an original, simple and economical tool to stimulate critical thinking on Social Determinants of Health, and to stimulate other skills. Photovoice can be considered in teaching about aspects related to health/care in Nursing students, in order to promote critical thinking of future agents for a change in health.

## Abstract (pt)

**Objetivo:**

explorar as potencialidades da metodologia Photovoice para estimular o pensamento crítico sobre os Determinantes Sociais da Saúde.

**Método:**

estudo exploratório e descritivo, com abordagem qualitativa, utilizando diferentes etapas da metodologia Photovoice. Os estudantes de enfermagem obtiveram fotografias em sua comunidade, mostrando Determinantes Sociais da Saúde, analisaram e classificaram as fotografias e expuseram os resultados na faculdade. Os alunos preencheram um questionário escrevendo suas percepções. Os dados coletados dos questionários foram analisados qualitativamente. **Resultados:** 91 estudantes participaram do estudo. Duas categorias principais emergiram dos dados: A Photovoice é uma boa metodologia para estimular o pensamento crítico sobre os determinantes sociais da saúde e estimular outras habilidades (expressar crenças e percepções, estimular a criatividade, desenvolver habilidades de pesquisa, fortalecer laços com colegas e atrair atenção).

**Conclusão:**

exploramos as potencialidades da metodologia Photovoice. Esta pode ser uma ferramenta original, simples e barata para estimular o pensamento crítico sobre os Determinantes Sociais da Saúde e estimular outras habilidades. A Photovoice pode considerar o ensino de aspectos relacionados à saúde/assistência em estudantes de enfermagem, a fim de promover o pensamento crítico de futuros agentes de mudança na saúde.

## Abstract (es)

**Objetivo:**

explorar las potencialidades de la metodología del Photovoice para estimular el pensamiento crítico sobre los Determinantes Sociales de la Salud.

**Método:**

estudio exploratorio y descriptivo con enfoque cualitativo, en el que se utilizaron diferentes pasos de la metodología del Photovoice. Los estudiantes de enfermería tomaron fotografías en su comunidad, en las que se mostraban los Determinantes Sociales de la Salud, las analizaron y clasificaron y expusieron los resultados en la facultad. A continuación, completaron un cuestionario escribiendo sus percepciones sobre las mismas. Los datos recopilados de los cuestionarios se analizaron cualitativamente.

**Resultados:**

en el estudio participaron 91 estudiantes. Surgieron dos categorías principales de los datos: Photovoice es una buena metodología para estimular el pensamiento crítico sobre los Determinantes Sociales de la Salud, y Photovoice es una buena metodología para estimular otras habilidades (expresar creencias y percepciones, estimular la creatividad, desarrollar habilidades de investigación, fortalecer los lazos con los compañeros y atraer la atención).

**Conclusión:**

exploramos las potencialidades de la metodología Photovoice. Puede ser una herramienta original, simple y económica para estimular el pensamiento crítico sobre los Determinantes Sociales de la Salud, y también para estimular otras habilidades. Photovoice se puede considerar para la enseñanza de aspectos relacionados con la salud y/o el cuidado con estudiantes de enfermería, para promover el pensamiento crítico de futuros agentes de cambio en la salud.

## Introduction

New strategies in the teaching and learning of Nursing students suggest the combination of traditional education, focused on acquiring knowledge and shaping behaviors, with methodologies in which dimensions such as values, beliefs, feelings, experiences, or the circumstances of the students, are integrated, as well as creativity and critical thinking^(^ 1 ^)^.

These new trends require that students participate and interact directly in the learning process, an important challenge for academics. Some of the objectives that these new teaching approaches aim to cover are the following: acquiring knowledge, bringing theoretical concepts closer to those of the care practice, and getting students to come to a better understanding of complex nature/complex phenomena such as those related to health and care^(^ 2 ^)^.

As professors, if we want to teach students in that direction, we must promote experiences related to health-disease and care, as well as promote critical dialogue.

In this way, qualitative techniques and methods, framed in the interpretative and critical paradigms, have been used in different ways in the academic field of our discipline to promote this reflection^(^ 2 ^-^ 3 ^)^. Photography has also been used in the reflective educational sphere^(^ 4 ^-^ 7 ^)^, in order to create images and form knowledge about the social sphere. In that sense, there are studies that have used participatory photography or Photovoice. Photovoice is defined as a participatory method that allows people to “identify, represent, and enhance their community through a specific photographic technique”^(^ 8 ^)^. This participatory photography has three main goals: i) to enable people to record and reflect their community´s strengths and concerns, ii) to promote critical dialogue and knowledge about important issues through large and small group discussions of photographs and iii) to reach policymakers^(^ 8 ^)^. Since its development in the 1990s, with research centered in contexts of critical consciousness and feminist theory, the method has been used in health education and related fields^(^ 9 ^-^ 12 ^)^. Nowadays, it has value as an educational tool with Nursing students^(^ 13 ^-^ 14 ^)^.

This research aims to explore the potentialities of the Photovoice methodology to stimulate critical thinking in Nursing students about factors that affect individual and community health.

Social Determinants of Health (SDHs) are “the conditions in which people are born, grow, work, live, and age, and the wider set of forces and systems shaping the conditions of daily life”^(^ 15 ^)^. These circumstances are shaped by the distribution of money, power, and resources at the global, national, and local levels. The World Health Organization (WHO) asserts that the SDHs are mostly responsible for health inequities, for the unfair and avoidable difference in health status seen within and between countries. The key concepts are employment conditions, social exclusion, public health programs and social determinants, women and gender equity, early child development, globalization, health systems, measurement and evidence or urbanization^(^ 15 ^)^. Global initiatives^(^ 16 ^-^ 17 ^)^ have considered these social factors in their programs, in order to eliminate health disparities.

Regarding the WHO recommendations for health professionals, it seems to be interesting to address SDHs in the Nursing curricula and to promote critical thinking about it. Some researchers have promoted this critical thinking about the importance of the social and cultural dimension of health through the simulation method^(^ 18 ^)^ or service learning^(^ 19 ^)^, for instance.

In this way, as professors involved in a subject in which SDHs are integrated in its curricula, we proposed to reinforce their contents through community observation, introducing qualitative methodologies to explore their perceptions and to promote critical dialogue.

We wonder if Photovoice will allow the students to narrate their experiences and produce knowledge about their context.

The objective of the following research was to explore the potentialities of the Photovoice methodology to stimulate critical thinking on Social Determinants of Health.

## Method

Design: An exploratory and descriptive study with a qualitative approach, using different steps of the Photovoice methodology (Photo-documentation, Photo-elicitation, and Exhibition in gallery)^(^ 12 ^)^.

Participants and Setting: The project was conducted from November 2018 to January 2019, in the Health Science School, León Campus, University of León (León, Spain). The University of León is a public university with approximately 10,200 students between degrees, Masters and Doctorates. The Nursing degree consists of 4 courses and has approximately 600 students.

For this teaching approach, all the students enrolled in the “Community Nursing” subject were invited to participate (convenience sampling). The subject takes place in the first semester of the second undergraduate year in Nursing.

Data collection: In a first session (2 hours), the concepts of the SDHs, the Photovoice methodology, and the project objectives were explained. The students were instructed to obtain photographs showing some of the factors, on a social level, that are decisive for the health of the people in their community. They had 15 days to obtain these photos (Photo-documentation), using their phones, tablets or cameras, and respecting the anonymity of the individuals who appeared in photographs (pixelating faces). They selected and printed 3 of these photographs and filled in 3 documents (one *per* photograph), titling each photograph, and answering the SHOWED mnemonic method. The SHOWED mnemonic method is composed by 5 questions: What do you See here? What is really Happening? How does this relate to Our lives? Why does this problem or strength Exist? What can we Do about it?^(^ 20 ^-^ 21 ^)^.

In a second session (2 hours), a participatory analysis of the data was carried out (Photo-elicitation). All the students were divided in 5 classrooms of approximately 20 students *per* class (91 students = 5 classes). Each classroom with 20 students was divided in groups (5 students *per* group). Firstly, in each group of 5, each student had to explain the 3 selected photographs to the other 4 students, using their SHOWED-based narratives. A discussion group was established, and they had to select the best 5 photographs that they thought best-reflected the SDHs of their community. Secondly, a discussion group was established between all the students of the classroom. They had to carry out a qualitative analysis of the photographs^(^ 8 ^)^, classifying them into categories. In order to identify the categories, the professor provided the students the WHO classification on SDHs. To ensure saturation of the data and of the categories emerged, the topics were compared and confirmed by all the groups of students.

Finally, a mural was constructed in each classroom, with the 20 photographs (1 mural *per* classroom = 5 murals) and all the murals were exhibited for a month in the hall of the Nursing school (Exhibition in gallery). Following recommendations of the Photovoice methodology^(^ 8 ^)^, this exhibition of images can promote critical debate in students community about SDHs, and invite to reflect on what they can do about it.

Considering that this research aimed to explore the potentialities of the Photovoice methodology in order to stimulate critical thinking, we created a questionnaire to be filled out by the students (paper). In it, they had to write their perceptions about this Photovoice experience.

The questionnaire was composed *ad-hoc*, and it consisted of 10 open questions, in order to know if Photovoice was perceived by the students as a good methodology related to stimulating reflective thinking and other skills. It was anonymous. The students had to complete it at the end of the second session.

The professor (IP) had an active role during the two sessions, promoting debate among the students and providing feedback in the discussion groups. She observed the participants, taking field notes of the relevant conversations and attitudes of the different discussion groups.

The data collected from the questionnaires were qualitatively analyzed manually^(^ 22 ^)^: i) pre-analysis, ii) exploration of the data, iii) treatment of the results by inference and interpretation. The meaningful units were searched, codified, and grouped into main categories, derived from the data. A qualitative matrix was elaborated, with categories, subcategories, and textual citations. The emerged categories were discussed between the IP and the students, and an external colleague (expert in qualitative analysis and in participatory methods with students) validated the data.

The notes obtained from the field notes (observation of the participants) and the photographs helped to triangulate the information obtained from the questionnaires and to confirm data saturation. The conclusions were shown to the students, in order to confirm them. The data obtained were relevant both in the concrete context and in others, comparing with other research studies. The results were validated as rigorous in the design, in the process to obtain the data, and in the interpretation. An attitude of self-criticism was maintained throughout the process, to achieve reflexivity.

Ethical aspects: An informed consent was requested from the students, allowing the use of their photographs and of their answers to the questionnaire for the research. The Ethics Committee of the University of León (ETICA-ULE-037-2018) approved the research.

## Results

All the students enrolled in the subject (a total of ninety-one) participated in the Photovoice experience and answered the questionnaire. They were from 19 to 37 years old, 71 of them were female, and 20 were men.

The results were obtained from a qualitative analysis of the questionnaires, of the notes obtained from the field notes (observation of the participants), and of the photographs.

**Figure 1:**
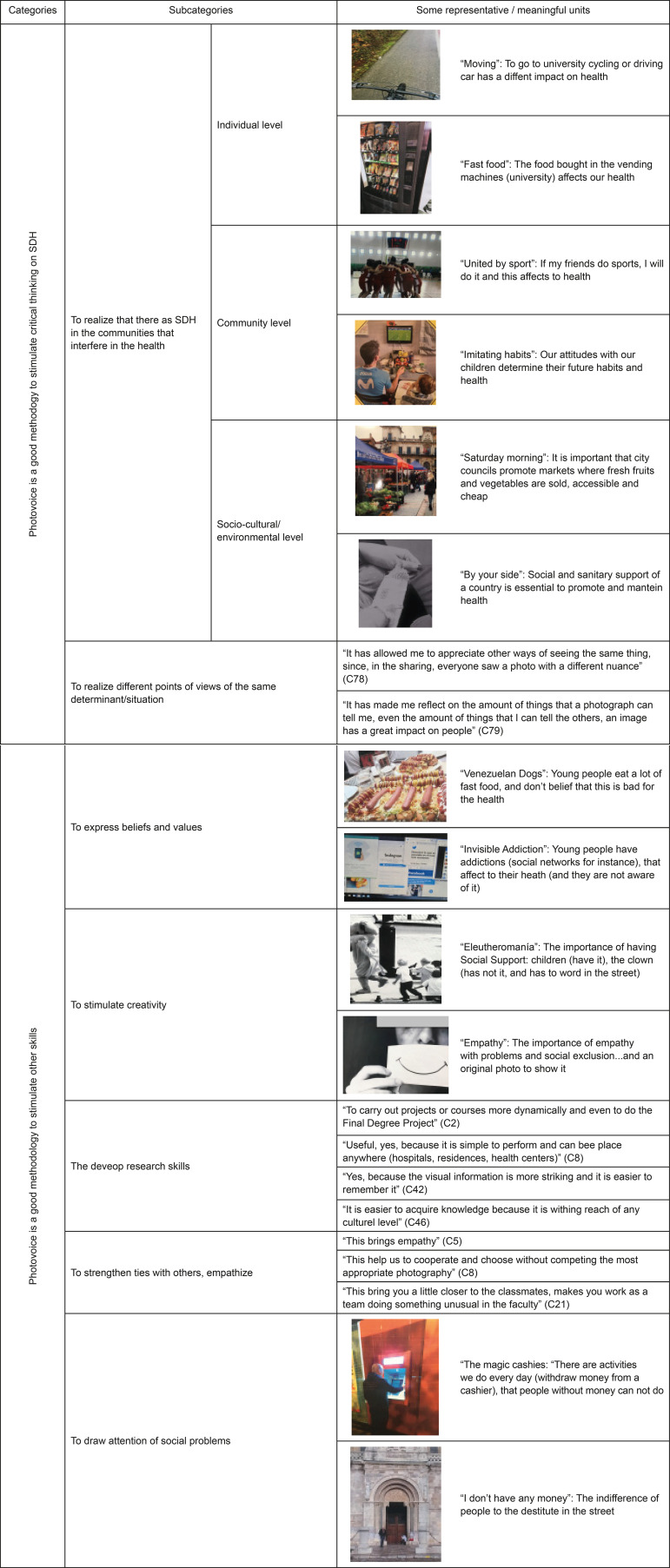
Categories, subcategories and some representative photographs/meaningful units emerged from the data

Two main categories emerged from the data: Photovoice is a good methodology to stimulate critical thinking on SDHs, and Photovoice is a good methodology to stimulate other skills. In Matrix 1 we can see the categories and subcategories emerged from the data. Moreover, we showed some representative photographs and meaningful units that emerged from the data, as examples (Figure 1).

Following the definition from the WHO, the students classified all the photographs in 10 categories, corresponding to employment conditions, social exclusion, public health programs and social determinants, women and gender equity, early child development, globalization, health systems, measurement, and evidence or urbanization. In this way, on one hand, they realized which the SDHs are in the communities that interfere in health. Moreover, all of the photographs were placed on an Individual, Community, or Socio-cultural/environmental level.

Some of them answered that thinking about the SDHs of their neighborhood, photographing, and discussing about them helped them to realize different daily situations that go unnoticed, but which are interfering in their health.

Situations such as riding a bicycle to the university (individual), practicing sports with a group of friends (community), or the importance or support and care in early life (environmental) were some of the situations photographed by the students.

C24, for example, wrote that *Thinking about what I could take a photograph of forced me to think about the large number of situations or events that negatively affect our health. Photographing these situations helped me to raise awareness of the problem (...) to offer a critical eye that facilitates access to situations of rank in which to think (...). It helps me to improve the capacity for critical and analytical debate.*

C26 said, *This helped me to realize that there are many factors that affect our health, some see them far away (social exclusion) although they may be closer than we think.*

C32, *I had to analyze my environment looking for the determinants of health, which often go unnoticed. This is useful because many times we only consider these things in the classroom and not outside.*

On the other hand, they realized the different points of view of the same determinant/situation.

For instance, C78 said, *It has allowed me to appreciate other ways of seeing the same thing, since, when sharing, everyone saw the same photo with a different nuance.*

C79 told, *It has made me reflect on the amount of things that a photograph can tell me, or even on the amount of things that I can tell the others, an image has a great impact on people.*

The students wrote that Photovoice allowed them to promote other skills, classified in five categories:

- To express beliefs and values: The students said that photographing their community and discussing in the classroom helped them to externalize beliefs and values. For example, showing usual habits in young people, habits which have a negative impact on their health.

C7 wrote, *This brings some ability to express feelings and sensations, which is something that is usually difficult.* And C8, *It helps us to empower ourselves and move forward with our ideas.*

- To stimulate creativity: In order to look for special photographs, the students had to stimulate creativity and imagination. *During the process of taking pictures I enjoyed a lot, thinking about what to do, how to do it, being as creative as possible. The fact of discussing and choosing the photos in class, with my group, has been very exciting (...) this is my experience (…) to think differently, it is a very creative work, and it requires seeing another vision of things* (C2); *An entertaining and artistic way to see the determinants of health since they have seen very original and inspiring images (...). It can be used to develop ingenuity and imagination* (C9).

- To develop research skills: Some students qualified the Photovoice methodology as interesting for their present and their future. During their studies, to develop projects, the final degree project for instance. In the future, it can be implemented in the community, because they were considered easy to do, and easy to explain to people (older individuals for instance, or regardless of their cultural level).

C2 said, *To carry out projects or courses more dynamically and even to do the Final Degree Project*; C8, *Useful, yes, because it is simple to perform and can be implemented anywhere (hospitals, residences, health centers)*; C42, *Yes, because visual information is more striking and easier to remember*”; And C46, *It is easier to acquire knowledge because it is within reach of any cultural level.*

- To strengthen ties with others, empathize: Having to choose and discussing photographs and situations helped them to strengthen ties with their classmates, to improve integration in class, and to be empathetic: *This brings empathy* (C5); *This helps us to cooperate and choose without competing the most appropriate photography* (C8); *This brings you a little closer to the classmates, makes you work as a team doing something unusual in the faculty* (C21); *It helps to encourage teamwork. In addition, it helps to improve the group’s relationship* (C25); *Greater complicity and great knowledge of how to work in a team* (C90).

- To draw attention of social problems: According to some students, showing the photographs to other students/community (school) was interesting in order to draw attention and to promote reflection. In addition, photography became a vehicle through which to report social problems. *People become aware of real life and of what influences their day to day. They do not realize it because they do it daily* (C56); *It is a very visual way of raising awareness among people that there are many harmful habits that are normalized in society* (C65); *I think in this way you can draw the attention of people and influence something to change their habits*” (C91).

## Discussion

We found some Nursing research highlighting the use of photography as a valid tool to encourage reflective learning experiences about aspects related to health^(^ 7 ^,^ 23 ^)^. Using photographs (not pre-existing photos, but photographs taken by students, as in our study), some authors^(^ 6 ^,^ 24 ^)^ concluded that participatory photography encourages Nursing students to critically interoperate about culture and values. In the same way, but explicitly using the Photovoice methodology, in other research study, the students took photographs and reflected on different concepts of Nursing care^(^ 13 ^)^.

The results of these studies are in line with those obtained in our study, showing how photographic fieldwork and the Photovoice methodology can be good tools to incorporate in Nursing practices in the classroom. The goal: to stimulate, in a participatory way, critical thinking about aspects related to health and care.

Regarding the use of photography as a tool to stimulate other skills like expressing beliefs and values, our results are in line with several studies, which mentioned how photography can help students to express perceptions, emotions, cultural competences, or to develop empathy or engagement^(^ 23 ^-^ 24 ^)^. Some authors^(^ 14 ^,^ 24 ^)^ described photography as a tool to stimulate creativity and imagination of the students as well. Regarding the use of these techniques to enhance links with other classmates and strengthen collaboration and relationships (as in our research), there are studies that also mentioned it^(^ 13 ^-^ 14 ^,^ 24 ^)^. Finally, in order to capture the attention of the community to promote reflection and changes, in line with our results, some research studies^(^ 14 ^)^ also highlighted how reflective photography by the students allowed encouraging the needs of the community.

Concerning the limitations of our research, the time to do the discussion groups (2 hours) might have conditioned the students’ perceptions on the usefulness of this method. In other way, the fact of collecting the perceptions through questionnaires could have limited the answers, because of the short time that the students had to write, and the reduced space in the paper. For this reason, as future lines of research, it would be interesting to develop this type of photographic fieldwork experiences with more sessions, and to collect students’ perceptions through discussion groups or interviews, apart from questionnaires. Moreover, it would be interesting to apply this Photovoice methodology to other themes related to health and care.

In relation to the contributions of this research to the advancement of Nursing knowledge: SDHs is a topic that is considered essential in health policies at a global level^(^ 16 ^-^ 17 ^)^. Therefore, as future nurses, and as future agents of health change, students must know it first-hand. The fact of using participatory methods in classroom with Nursing students is a good and different way to stimulate critical thinking on SDHs^(^ 18 ^-^ 19 ^)^. This Photovoice experience with SDHs was original and novel: students using cameras/mobiles, reflecting both in the classroom and out of the classroom, participating in discussion groups, and cooperating to select the best photos, enriching themselves with the opinions of others, and constructing murals in order to draw attention and to promote the reflection of their community about SDHs. Photography is a usual tool that young people use daily to communicate and express themselves (Instagram or WhatsApp, for example). For this reason, regarding the Photovoice methodology, the fact of introducing tools as photography in teaching, considered common and attractive to young students, together with the simplicity of its realization, and its low cost (for students and professors alike), can encourage professors to introduce the Photovoice methodology in the Nursing academic field, in themes so current as SDHs. Something that has been considered only discreetly in teaching about health/care^(^ 12 ^,^ 14 ^,^ 24 ^)^.

## Conclusion

We explore the potentialities of the Photovoice methodology. Photovoice is an original, attractive, simple, and economical tool to stimulate critical thinking on SDHs. Moreover, this methodology can stimulate other skills. The Photovoice methodology can be considered in teaching about aspects related to health/care in Nursing students, in order to promote critical thinking of the future agents of health change.
